# Copper Transporter 2 Content Is Lower in Liver and Heart of Copper-Deficient Rats

**DOI:** 10.3390/ijms11114741

**Published:** 2010-11-19

**Authors:** Jesse Bertinato, Sébastien Duval, Mary R. L’Abbé

**Affiliations:** 1 Nutrition Research Division, Health Products and Food Branch, Health Canada, Sir Frederick G. Banting Research Centre, 251 Sir Frederick Banting Driveway, Ottawa, Ontario, K1A 0K9, Canada; E-Mail: drduby@hotmail.com; 2 Department of Nutritional Sciences, Faculty of Medicine, University of Toronto, FitzGerald Building, 150 College Street, Toronto, Ontario, M5S 3E2, Canada; E-Mail: mary.labbe@utoronto.ca

**Keywords:** Ctr2, copper, rat, diet, expression

## Abstract

Copper (Cu) transporter 2 (Ctr2) is a transmembrane protein that transports Cu across cell membranes and increases cytosolic Cu levels. Experiments using cell lines have suggested that Ctr2 expression is regulated by Cu status. The importance of changes in Ctr2 expression is underscored by recent studies demonstrating that lower Ctr2 content in cells increases the cellular uptake of platinum-containing cancer drugs and toxicity to the drugs. In this study, we examined whether Ctr2 expression is altered by a nutritional Cu deficiency *in vivo*. Ctr2 mRNA and protein in liver and heart from rats fed a normal (Cu-N), moderately deficient (Cu-M) or deficient (Cu-D) Cu diet was measured. Rats fed the Cu-deficient diets showed a dose-dependent decrease in liver Ctr2 protein compared to Cu-N rats. Ctr2 protein was 42% and 85% lower in Cu-M and Cu-D rats, respectively. Liver Ctr2 mRNA was 50% lower in Cu-D rats and unaffected in Cu-M rats. In heart, Ctr2 protein was only lower in Cu-D rats (46% lower). These data show that Cu deficiency decreases Ctr2 content *in vivo*.

## 1. Introduction

Copper (Cu) transporter 1 (Ctr1) and Cu transporter 2 (Ctr2) are structurally similar Cu transporters that transfer Cu across cell membranes and increase Cu levels in the cytosol of cells. Ctr1 is a major Cu importer that mediates high affinity Cu uptake at the plasma membrane in the form of a homotrimer [1–4]. Less is known about the function and structure of Ctr2; however, Ctr2 functions as a homomultimer similar to Ctr1 [5,6]. In the yeast *Saccharomyces cerevisiae*, Ctr2 localizes to the vacuole and transports Cu from the vacuole to the cytosol [6]. A similar role has been described for the *Schizosaccharomyces pombe* ortholog Ctr6 [7]. In mammalian cells, Ctr2 has been localized in late endosomes and lysosomes [5] and the cell nucleus [8]. Our lab has shown that in African green monkey kidney COS-7 cells, Ctr2 localizes predominantly to large cytoplasmic vesicles with a small proportion distributed at the cell surface where it transports Cu into cells [9].

It is now well-established that proteins involved in the trafficking of Cu also play an important role in the metabolism of commonly used platinum-based chemotherapeutic drugs such as cisplatin and carboplatin [10–18]. Mouse embryonic fibroblast (MEF) cells lacking Ctr1 showed a marked reduction in cisplatin accumulation and increased resistance to its cytotoxic effects [12]. In contrast to Ctr1, lower levels of Ctr2 increases the cellular uptake of platinum-based chemotherapeutic drugs and toxicity to the drugs [16,17]. Knocking down Ctr2 in MEF cells increased the accumulation of cisplatin by 2–3-fold, which was associated with increased cytotoxicity to cisplatin [16]. Furthermore, an evaluation of several human ovarian carcinoma cell lines revealed that Ctr2 content was inversely correlated with sensitivity to cisplatin [16].

A recent study by Blair *et al.* has suggested that lower Ctr2 content in cells increases the uptake of cisplatin by increasing the rate of macropinocytosis through activation of Rac1 and cdc42 [17]. In that study, Blair *et al.* also showed that, in mice, tumors formed from cells in which Ctr2 was knocked down using a lentiviral vector expressing a shRNAi directed against Ctr2 grew significantly slower compared to tumors formed from the parental cell line [17]. Tumors from Ctr2 knockdown cells showed increased frequency of apoptotic cells and decreased vascular density.

Cellular Cu homeostasis is maintained by regulating the uptake, intracellular distribution and elimination of Cu via the activity of Cu transporters and chaperones [19]. Changes in cellular expression of Cu transporters and chaperones in response to changes in Cu availability are likely regulatory mechanisms that have evolved to help cells cope with sub- or supra-optimal levels of Cu. We and others have shown that Cu promotes the degradation of Cu chaperone for Cu/Zn superoxide dismutase (CCS) [20,21]. Cu binding to the *C*-terminal CXC (C, cysteine; X, any amino acid) Cu-binding motif decreases CCS stability and increases the rate of degradation by the 26 S proteasome [20,21]. Induction of Cu deficiency in animals by feeding a Cu-deficient diet increases CCS content in tissues and therefore CCS is a good indicator of Cu deficiency *in vivo* [20,22–25]. Elevated CCS under conditions of Cu deficiency may increase the efficiency of Cu delivery to its target enzyme Cu/Zn superoxide dismutase (SOD1) and preserve its activity in the cell. Expression of Ctr1 is also regulated by Cu availability. Addition of supplemental Cu to certain cell types induces the internalization of Ctr1 from the plasma membrane and degradation of the protein [26]. Ctr1 content is also increased in tissues of Cu-deficient mice [27,28]. Changes in Ctr1 expression may be a regulatory mechanism to reduce cellular Cu uptake when Cu is in excess and increase uptake when Cu is scarce.

*In vitro* experiments using cell lines have suggested that Ctr2 expression is regulated by Cu status [8]. Ctr2 mRNA and protein in human ovarian carcinoma 2008 and MEF cells increased when cells were cultured in medium supplemented with a high concentration of Cu (*i.e.*, 200 μmol/L) [8]. Conversely, Ctr2 mRNA and protein were decreased in cells cultured in medium supplemented with the Cu chelator bathocuproine disulfonate [8]. In cells treated with cyclohexamide to block protein synthesis, Ctr2 protein showed an extended half-life when incubated in the presence of supplemental Cu, suggesting that Cu status regulates Ctr2 expression, at least in part, by a post-translational mechanism [8]. Interestingly, post-transcriptional regulation of Ctr2 in response to Cu deficiency or excess appears to be dependent on the Cu chaperone ATOX1 [8].

Given the *in vitro* evidence suggesting that Cu status determines Ctr2 content in cells, we investigated whether Cu deficiency affects Ctr2 expression *in vivo*. In this study, we show that Ctr2 content is decreased in a dose-dependent manner in liver and heart of rats with a nutritional Cu deficiency.

## 2. Results and Discussion

In this study we examined whether Ctr2 mRNA and protein are altered in response to Cu deficiency in liver and heart of rats. Rats were fed a Cu-normal (Cu-N), moderately Cu-deficient (Cu-M) or Cu-deficient (Cu-D) diet. We have previously characterized the Cu status of these rats [22]. Rats fed the Cu-restricted diets showed changes in established biomarkers of Cu deficiency. Liver and erythrocytes had decreased activity of the Cu-dependent enzyme SOD1 and increased expression of its chaperone CCS indicating that the rats had depressed Cu status [22]. Notably, CCS content in liver and erythrocytes of Cu-D rats was higher compared to Cu-M rats indicating more pronounced Cu deficiency in Cu-D rats [22].

Ctr2 content in liver and heart extracts was measured by Western blot using a custom made Ctr2 antibody. Our group and others have previously used this antibody to detect transiently overexpressed Ctr2 and endogenous Ctr2 in rat tissues and cell lines from different species [8,9,16,17]. Our previous work showed that our Ctr2 antibody detected a predominant band of ∼70 kDa in size in COS-7 cells and rat tissues, including liver and heart [9]. Because the primary amino acid sequence of Ctr2 predicts a protein of ∼17 kDa, it was important to establish that the 70 kDa band detected represents Ctr2. We previously showed that in COS-7 cells, siRNAs targeted to different regions of the Ctr2 mRNA sequence were able to knockdown the 70 kDa band detected with our Ctr2 antibody confirming that the 70 kDa band represents Ctr2 [9]. Moreover, Blair *et al.* have also detected Ctr2 as a ∼70 kDa band in MEF cells [17]. However, in contrast with our data they also detected a band of ∼17 kDa of about equal intensity. The intensities of both the 70 and 17 kDa bands were strongly depressed in cells infected with lentivirus expressing a shRNAi targeting the mouse Ctr2 mRNA confirming that both bands represent Ctr2. The 17 kDa band represents the Ctr2 monomer as this is the predicted size based on the amino acid sequence of Ctr2. Given that Ctr2 functions as a homomultimer, the 70 kDa band likely represents a mutimeric form of Ctr2. It is unclear why we did not detect the 17 kDa band in COS-7 cells and rat tissues, but it may be explained by species differences or differences in the preparation of the protein extracts or Western blotting procedure that affects the mutimerization state of Ctr2.

**Figure 1.**
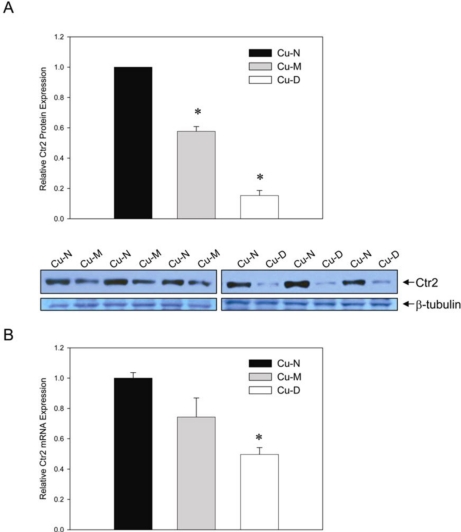
Ctr2 expression in liver of Cu-deficient rats. (**A**) Representative Western blot of liver extracts from Cu-N, Cu-M or Cu-D rats probed with an antibody against Ctr2. The membrane was stripped and probed with an antibody against β-tubulin. Intensities of the Ctr2 bands relative to β-tubulin are depicted graphically (*top panel*); (**B**) QPCR analyses of Ctr2 mRNA expression in liver. Ctr2 mRNA abundance is presented relative to GAPDH mRNA abundance. For Cu-N rats, Ctr2 expression was arbitrarily set to 1. Values are means ± SEM, *n* = 3 (**A**) or *n* = 6 (**B**). * Different from Cu-N rats, *P* < 0.05.

Consistent with our previously published data, Ctr2 was detected as a single strong band of ∼70 kDa in size in liver and heart extracts. Ctr2 protein showed a dose-dependent decrease in liver of rats fed diets low in Cu (Figure 1A). In comparison to Cu-N rats, Ctr2 was 42% lower in Cu-M rats and showed a marked 85% reduction in Cu-D rats (Figure 1A). A repetition of the experiment using three different rats from each diet group gave similar results (data not shown). Further, all Cu-D rats from this study showed a marked reduction in liver Ctr2 protein content (data not shown). The observed magnitude of change in Ctr2 is significant considering that a modest 33–55% reduction in Ctr2 protein in MEF cells in knockdown experiments resulted in a 2–3-fold increase in the accumulation of the platinum-based chemotherapeutic drug cisplatin, which was associated with a large increase in toxicity of the drug [16].

Whereas Ctr2 protein was markedly lower in liver of Cu-restricted rats, Ctr2 mRNA was less affected. Liver Ctr2 mRNA was not significantly lower in Cu-M rats compared to Cu-N rats (Figure 1B). In Cu-D rats, Ctr2 mRNA was 50% lower compared to Cu-N rats (Figure 1B). The larger magnitude of decrease of Ctr2 protein compared to mRNA indicates that both transcriptional and post-transcriptional mechanisms regulate Ctr2 expression in response to decreased Cu status in rat liver. These results are in agreement with the cell culture experiments of Blair *et al*. demonstrating both transcriptional and post-translational regulation of Ctr2 expression in response to addition of high amounts of Cu or a Cu chelator to the cell culture medium [8]. The decrease in Ctr2 protein in the absence of a significant decrease in Ctr2 mRNA in Cu-M rats with a milder Cu deficiency suggests that the post-transcriptional mechanism for Ctr2 regulation is more sensitive to moderate reductions in Cu status compared to the transcriptional mechanism. Thus, the post-transcriptional mechanism is likely the more biologically relevant mechanism for Ctr2 regulation in response to Cu deficiency. It is noteworthy to mention that human ovarian carcinoma 2008 cells cultured in the presence of a Cu chelator showed a modest 29% decrease in Ctr2 mRNA, but a much larger 90% decrease in Ctr2 protein [8].

Ctr2 protein in heart of Cu-M rats was not significantly lower compared to Cu-N rats (1.00 ± 0.12 *vs.* 0.86 ± 0.30, *P* > 0.05, *n* = 4). However, heart Ctr2 protein was lower in Cu-D rats (1.00 ± 0.03 *vs.* 0.54 ± 0.12, *P* < 0.05, *n* = 4). Compared to liver Ctr2, the magnitude of decrease in heart Ctr2 was smaller indicating that Ctr2 expression in liver is more sensitive to decreases in Cu status in rats.

It is presently not understood why Ctr2 content is reduced in response to Cu deficiency. It has been demonstrated that knocking down Ctr2 increases Cu uptake in MEF cells expressing Ctr1, but not in an isogenic cell line lacking Ctr1 [16]. These data suggest that a decrease in Ctr2 increases cellular Cu uptake directly by Ctr1 or by a Ctr1-dependent mechanism. It is possible that lower Ctr2 content in cells may act as a signal of low intracellular Cu levels that stimulates Cu uptake by the cell.

## 3. Experimental Section

### 3.1. Animals and Test Diets

The experimental animal protocol and test diets have been described elsewhere [22]. Briefly, weanling (21-day-old) male Wistar rats (Charles River Canada, St. Constant, Canada) were randomly assigned to 1 of 3 diet groups (*n* = 10/group). Test diets were nutritionally complete but contained different amounts of Cu. Cu-normal (Cu-N), moderately Cu-deficient (Cu-M) or Cu-deficient (Cu-D) diets contained 5.3 ± 0.06, 0.84 ± 0.03 and 0.34 ± 0.01 mg of Cu/kg diet, respectively. After 6 weeks of feeding the diets, rats were killed and tissues were collected and stored at −80 °C until analysis. The experimental protocol was approved by the Animal Care Committee of the Health Products and Food Branch of Health Canada.

### 3.2. Tissue Extracts and Western Blotting

Preparation of tissue extracts and Western blotting procedure were performed essentially as described [9]. Liver and heart were homogenized in 0.5% Triton X-100 supplemented with a protease inhibitor cocktail (Roche Diagnostics, Laval, Canada). Supernatant was recovered following centrifugation and total liver (40 μg) or heart (20 μg) proteins were separated over 8–16% Tris-Glycine gradient gels (Invitrogen, Burlington, Canada). Proteins were electroblotted onto nitrocellulose membranes. Membranes were incubated with a primary antibody against Ctr2 that we have described previously [9] or an antibody against β-tubulin (H-235, Santa Cruz Biotechnology, Santa Cruz, CA) for 3 h at room temperature at a concentration of 2 μg/mL or 0.67 μg/mL, respectively. Following incubation with an anti-rabbit horseradish peroxidase-conjugated secondary antibody, proteins were detected by enhanced chemiluminescence and exposure to film. Film was scanned and band intensities were determined using Scion Image software (Scion Corporation, Frederick, MD).

### 3.3. Quantitative PCR (QPCR)

Total RNA was extracted from liver using the RNeasy^®^ kit (QIAGEN, Mississauga, Canada) and cDNA was generated using an oligo(dT) primer. QPCR was performed with an Mx4000 Multiplex Quantitative PCR System (Stratagene, La Jolla, CA) using the Brilliant SYBR Green QPCR reagent kit (Stratagene) and primer sets specific for Ctr2 (forward primer, 5’-AACTTCAGACAATAGGACCCGCCT-3’; reverse primer, 5’-TAGGACATGACAGCCAGCATCACA-3’) or glyceraldehyde-3-phosphate dehydrogenase (GAPDH) (forward primer, 5’-TCAAGAAGGTGGTGAAGCAGCC-3’; reverse primer, 5’-GCATCAAACGTGGAAGAATGGG-3’). Relative mRNA levels were determined using the standard curve method.

### 3.4. Statistical Analyses

Statistical analyses were performed using Statistica 8 software (StatSoft, Tulsa, OK). A *t*-test was used for comparison of means of 2 groups and an ANOVA test followed by a Dunnett test was used to compare means to a control group. Differences were considered significant at *P* < 0.05. Results were expressed as means ± SEM.

## 4. Conclusions

In this study, we have demonstrated that a nutritional Cu deficiency decreases Ctr2 content in liver and heart of rats. These results add biological relevance to recently published cell culture experiments suggesting that Ctr2 expression is regulated by Cu status [8,17]. Extending these *in vitro* observations to animals is particularly significant given the recent discoveries that lower Ctr2 content increases cellular uptake of commonly used platinum-based chemotherapeutic drugs, increases sensitivity to these drugs and slows tumor growth [16,17]. Given that Cu deficiency induces a number of adverse effects including anemia and impaired immune function [29–35], strategies aimed at lowering tumor cell Ctr2 levels by targeted reduction of Cu in tumor cells would be a better approach as opposed to depressing whole-body Cu status. Recently, nanoparticles have been engineered with the capability of targeted delivery of cisplatin to prostate cancer cells [36]. A similar approach could be envisioned for delivery of Cu-chelating drugs to cancer cells. With an efficient targeted delivery system, whole-body Cu status and Cu-dependent physiological processes would be expected to be minimally affected.
